# Concomitant Presence of Spermatic Cord and Testicular Non-Hodgkin's Lymphoma With Recurrence: A Case Report on a Rare Entity

**DOI:** 10.7759/cureus.30182

**Published:** 2022-10-11

**Authors:** Ganesh Panneerselvam, Gurubharath Ilangovan, Ealai A Parthasarathy, Rajamani Anand, Jeffrey S Joseph, Alam Khalil-Khan

**Affiliations:** 1 Department of Radiology, Chettinad Hospital and Research Institute/CARE, Chennai, IND; 2 Department of Academic Unit of Primary Medical Care, The University of Sheffield, Sheffield, GBR

**Keywords:** ptnhl, case report, relapse, non-hodgkin’s lymphoma, diffuse large b cell lymphoma, heterogeneously hypogenous mass, elderly male, inguino-scrotal swelling

## Abstract

Primary testicular non-Hodgkin’s lymphoma (PTNHL) with contiguous involvement of the spermatic cord is a rare occurrence and presentation of the disease, and it mostly involves elderly men between the sixth and eighth decades of life. PTNHL is a rare form of primary testicular malignancy that accounts for 1% of all non-Hodgkin's lymphoma cases and 5-10%of all testicular malignancies.

This case report discusses a 73-year-old man who presented with right-sided inguinoscrotal swelling for six months, which had progressively increased in size. The patient was referred to the surgical department, and the examination revealed a hard-palpable mass with thickening of the cord. The initial imaging included an ultrasound, demonstrating a heteroechoic mass inseparable from the right testis with evidence of mild increased internal vascularity. Due to the high suspicion of malignancy, a right orchidectomy was performed. The patient subsequently developed another swelling after seven months, over the right inguinal region, which had progressively increased in size. MRI of the pelvis and CT of the abdomen and chest revealed a lobulated, intermediate intense lesion in the right inguinoscrotal region.

This case report demonstrates the importance of radiological imaging in assessing and detecting the characteristics of concomitant lesions by using various imaging modalities and assessing the extent of spread. In addition, radiological imaging helps in the early diagnosis of the disease and facilitates prompt and early treatment to achieve favorable outcomes for the patient. The radiologist should include a differential diagnosis of PTNHL when imaging for a painless inguinoscrotal mass.

## Introduction

Lymphoma can initially present within the testis, and primary testicular non-Hodgkin’s lymphoma (PTNHL) and primary spermatic cord lymphoma (PSCL) are rare variants of gonadal tumors [1]. Both of these tumors have similar biological characteristics and share some imaging features. The concomitant presence of both tumors synchronously is indeed a rare phenomenon.

In this report, we aim to use the imaging features to identify, detect, stage, and assess the extent of the disease, and to help differentiate it from other gonadal tumors. The ultrasound plays an important role as the initial and basic modality to visualize the tumor followed by enhanced imaging modalities that aid in the diagnosis and thus benefit the patient. The risk factors include a history of cryptorchidism, recurrent orchitis, and trauma; however, these risk factors are not established entities, and the only risk factor that has a strong association is an HIV infection [2].

## Case presentation

**Figure 1:**
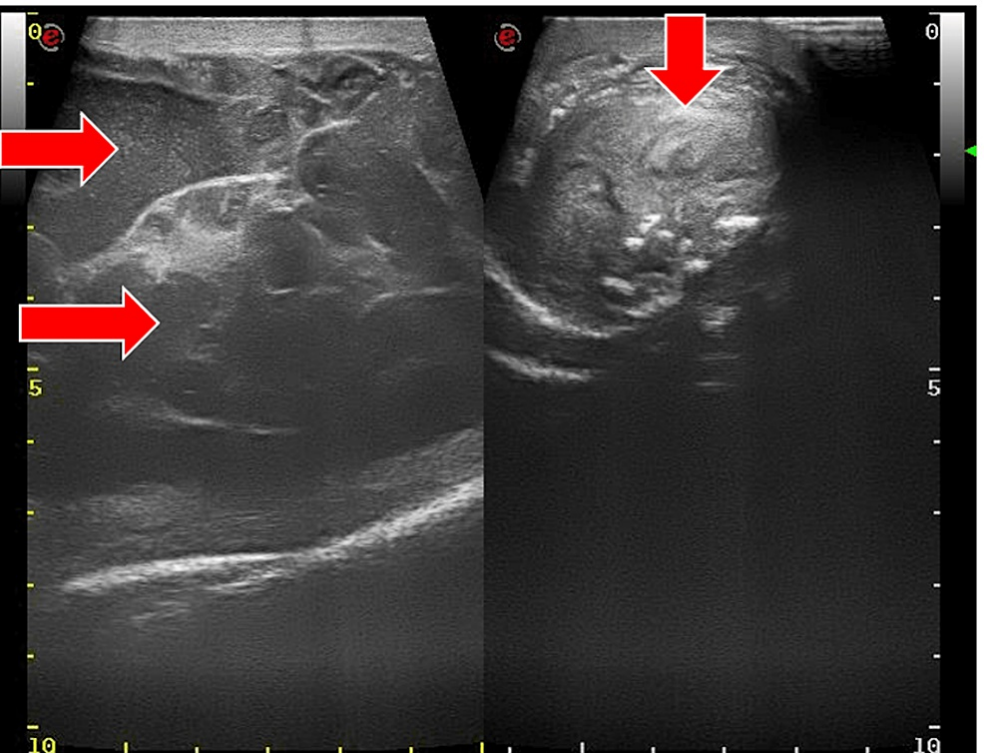
Ultrasound: scrotum - image 1 The image demonstrates heteroechoic mass that is inseparable from the right testis with evidence of a mild amount of echogenic free fluid surrounding the mass lesion

**Figure 2:**
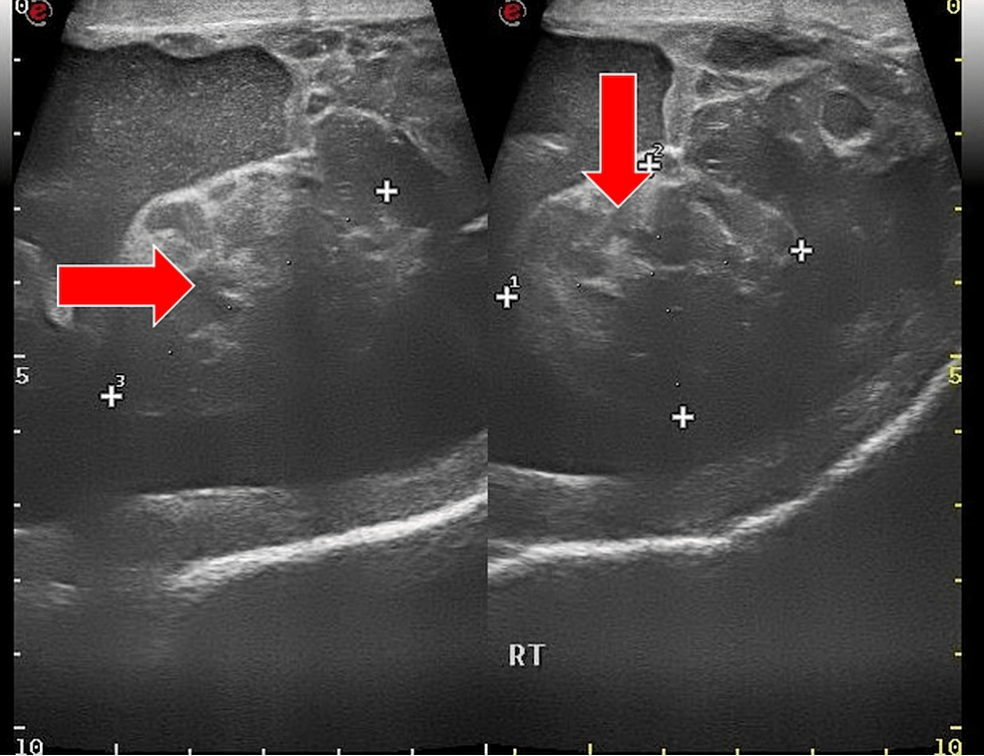
Ultrasound: scrotum - image 2 The image demonstrates heteroechoic mass that is inseparable from the right testis with evidence of a mild amount of echogenic free fluid surrounding the mass lesion

**Figure 3:**
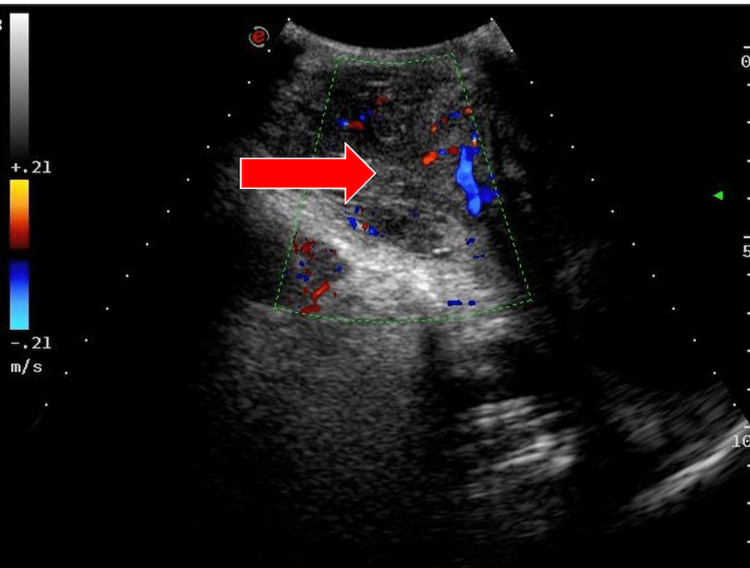
Ultrasound: scrotum - image 3 The image demonstrates increased and disorganized vascularity on color Doppler within the mass lesion

A 73-year-old man presented with a six-month history of right-sided inguinoscrotal painless swelling. The patient was referred to the surgical outpatient department. Examination findings revealed a hard palpable mass of 10 x 5 cm with thickening of the cord; the patient was subsequently referred for an ultrasound, which demonstrated a heteroechoic mass of 4.6 x 4.0 x 3.5 cm (Figures 1, 2) that was inseparable from the right testis, with evidence of internal vascularity (Figure 3). The cord structures were thickened and heterogenous in echotexture.

**Figure 4:**
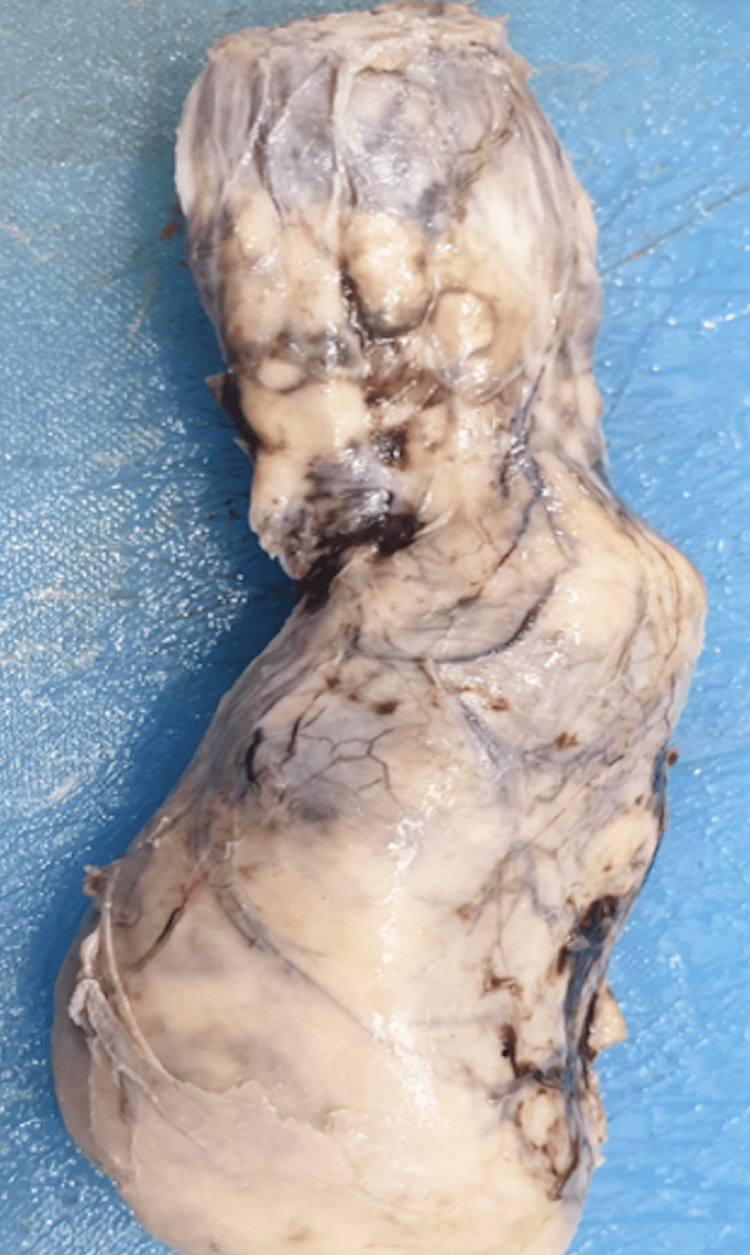
Gross specimen of enlarged testicular mass with enlarged and thickened spermatic cord structure The gross specimen shows an enlarged testicular mass measuring 9 x 4.5 cm with an enlarged and thickened cord structure measuring 5.5 x 4 cm; the external surface shows congested blood vessels with grey-brown areas

**Figure 5:**
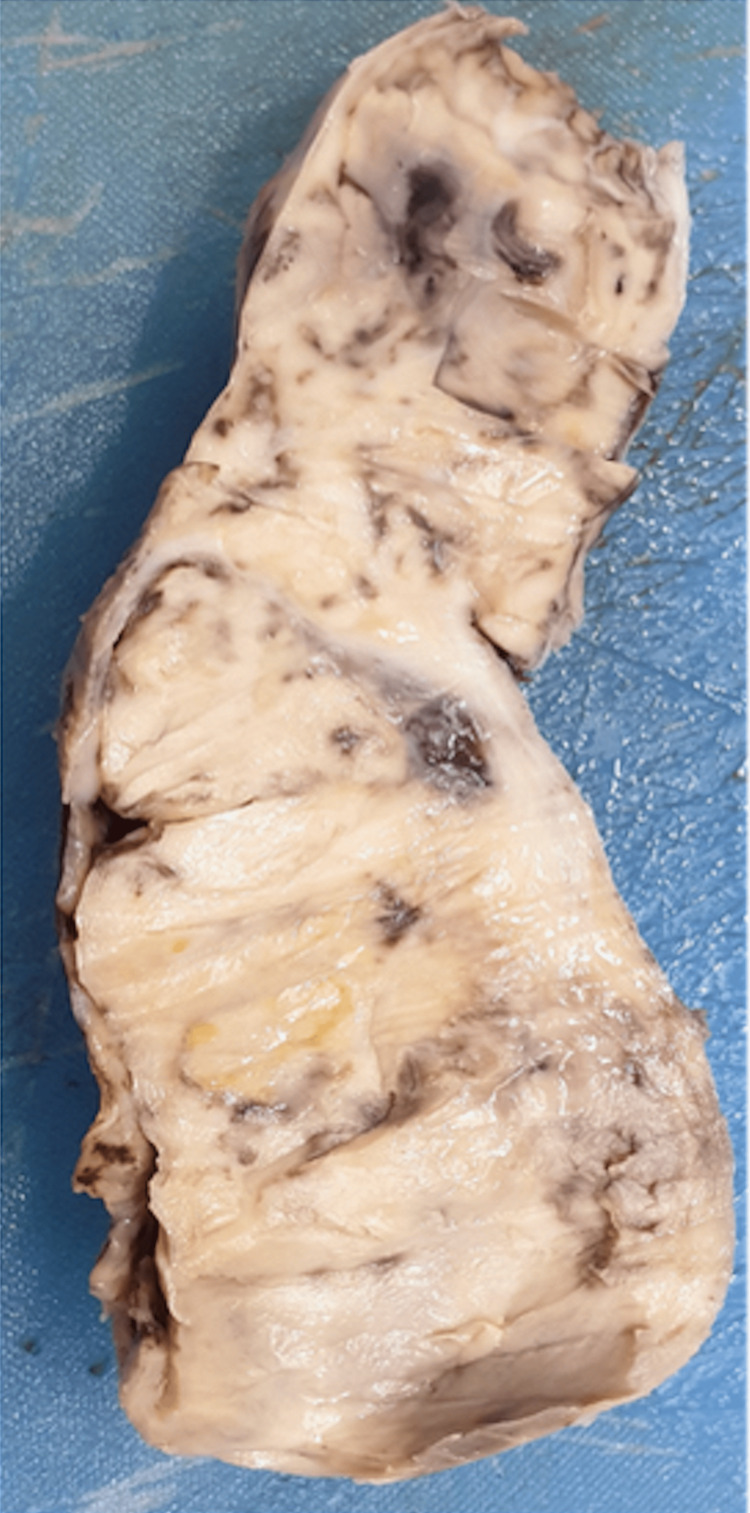
The cut surface of the spermatic cord The cut surface of the spermatic cord is replaced by grey-white to grey areas, some of which appear nodular along with grey-brown areas

**Figure 6:**
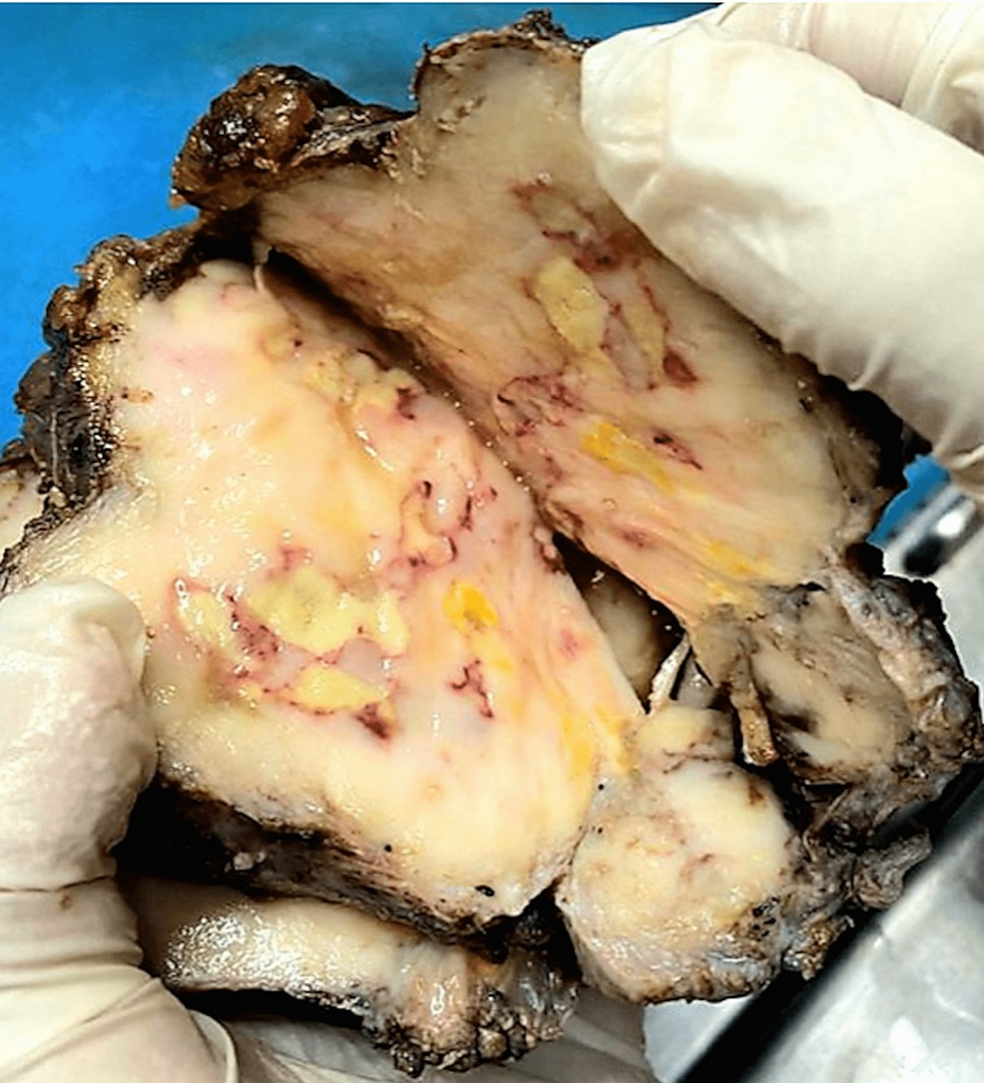
The cut surface of the testis The cut surface of the testis is replaced by grey-white to grey areas, some of which appear nodular along with grey-brown areas; no viable testicular area can be identified

An initial diagnosis of a mass of neoplastic origin was made and initial management involving right-sided low-level orchidectomy was performed. Intraoperatively, surgeons found a hard mass entirely replacing the right testis and epididymis measuring approximately 8 x 5 cm, with a thickened and nodular cord up to the level of the superficial inguinal ring (Figures 4-6).

**Figure 7:**
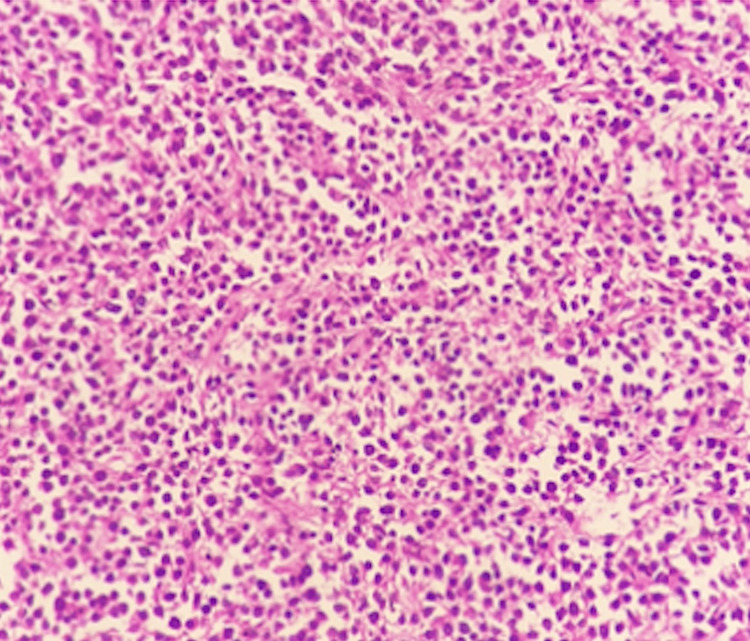
Haematoxylin & eosin staining of atypical cells The image demonstrates testis and spermatic cord tumor composed of diffuse sheets and discrete medium to large atypical cells with pale eosinophilic to clear cytoplasm, vesicular nuclei, prominent nucleoli, and increased mitosis. There are large areas of necrosis. 20x magnification

**Figure 8:**
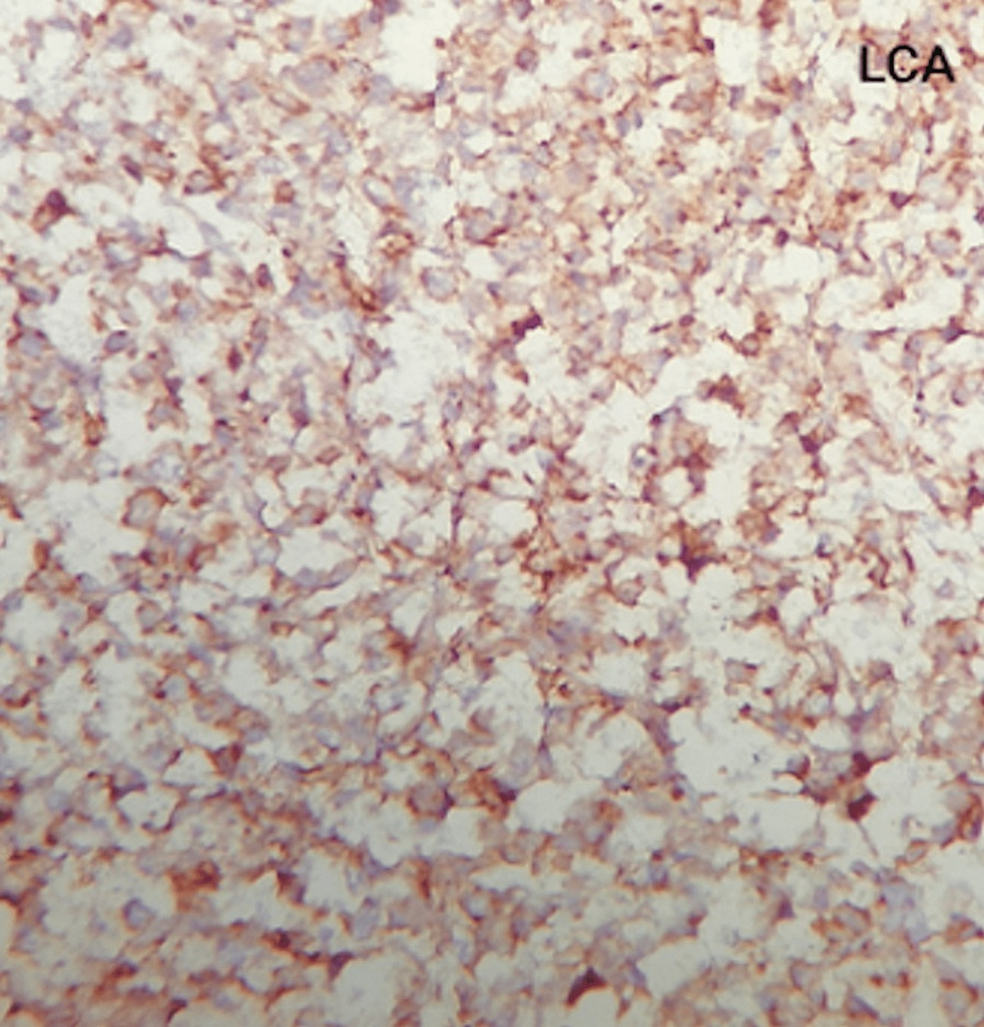
Immunohistochemistry stain (LCA) The image demonstrates diffuse membrane and cytoplasmic positivity for leukocyte common antigen (CD45). 40x magnification

**Figure 9:**
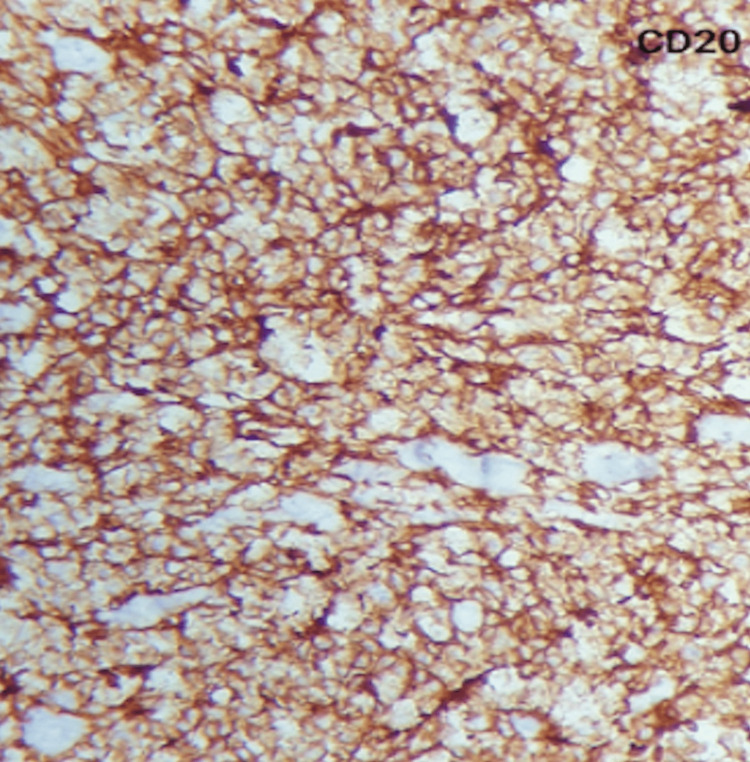
Immunohistochemistry stain (CD20) The image demonstrates diffuse membrane and cytoplasmic positivity for CD20. 40x magnification

The samples were sent for histopathological studies within the pathology department, which revealed diffuse sheets and discrete medium to large atypical cells with pale eosinophilic to clear cytoplasm, vesicular nuclei, prominent nucleoli, and increased mitosis (Figures 7-9). The tumor cells were seen to be encroaching on the local blood vessels. Similar types of cells were also seen in the ipsilateral spermatic cord. The samples were subjected to immunohistochemistry studies, which returned positive for cluster of differentiation 45 (CD45) also known as leukocyte common antigen (Figure 8), and positive (Figure 9) for cluster of differentiation 20 (CD20); these immunohistochemistry markers are specific for diffuse B-cell lymphoma. In the final report, a diagnosis of diffuse large B-cell type lymphoma was documented.

**Figure 10:**
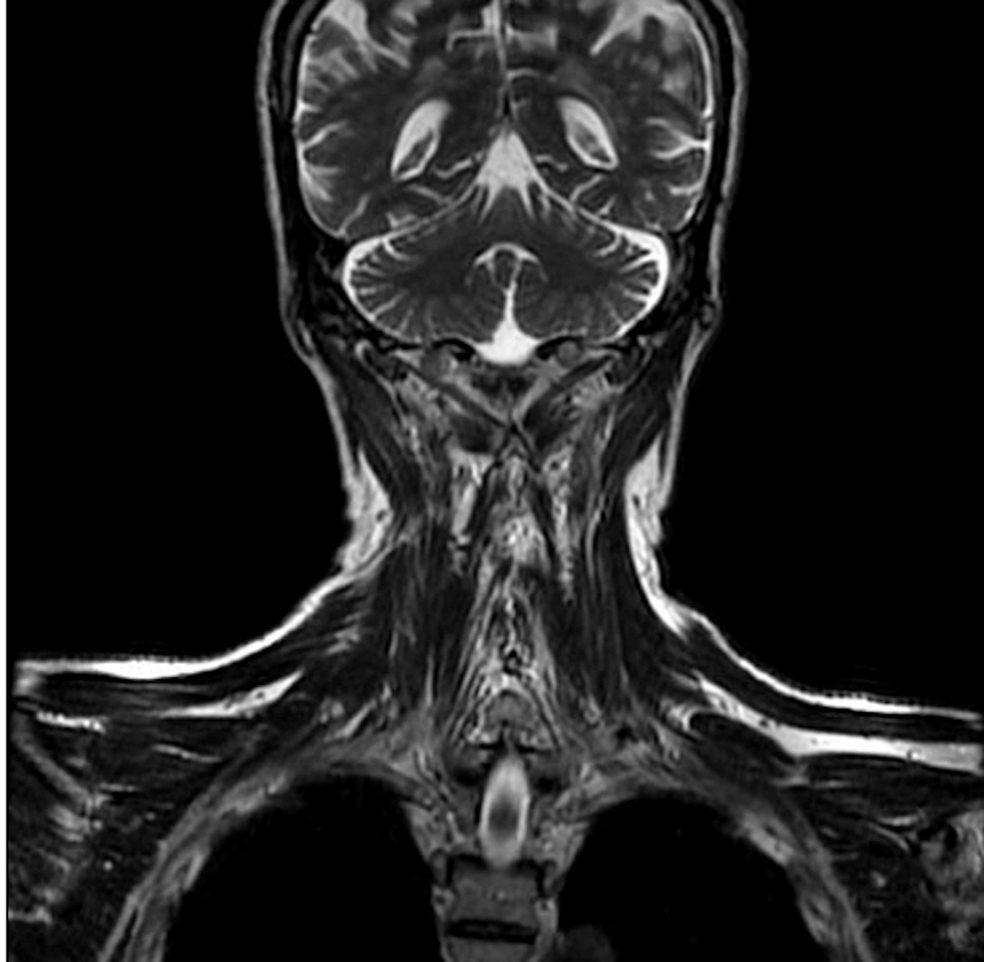
MRI head and neck T2-weighted MRI of head and neck coronal sections showed no discrete lesions MRI: magnetic resonance imaging

**Figure 11:**
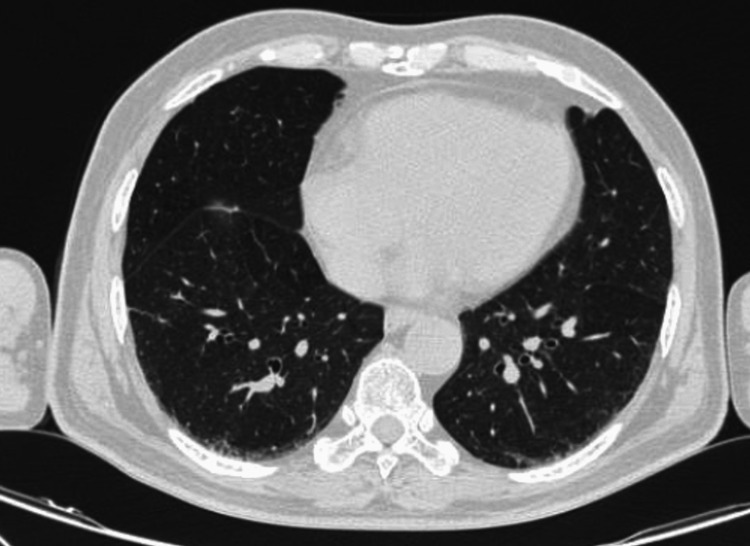
CT lung CT lung thin sections axial view showing no discrete lesions CT: computed tomography

Further imaging, including CT and MRI, was undertaken to investigate any spread of the disease. Imaging of the following regions was carried out: an MRI of the head and neck (Figure 10) and a CT thorax (Figure 11). The results revealed no spread within these typical regions where the disease can potentially spread. A further management plan was devised, which involved at least six cycles of chemotherapy; however, due to affordability issues for the patient and convenience factors, the patient was given six cycles of chemotherapy in the form of an R-CHOP regimen, which includes rituximab, cyclophosphamide, doxorubicin, vincristine, and prednisone; this was provided at the government-funded higher clinical center. Once the patient completed this part of the treatment, he was further advised to undergo radiotherapy, which he declined.

**Figure 12:**
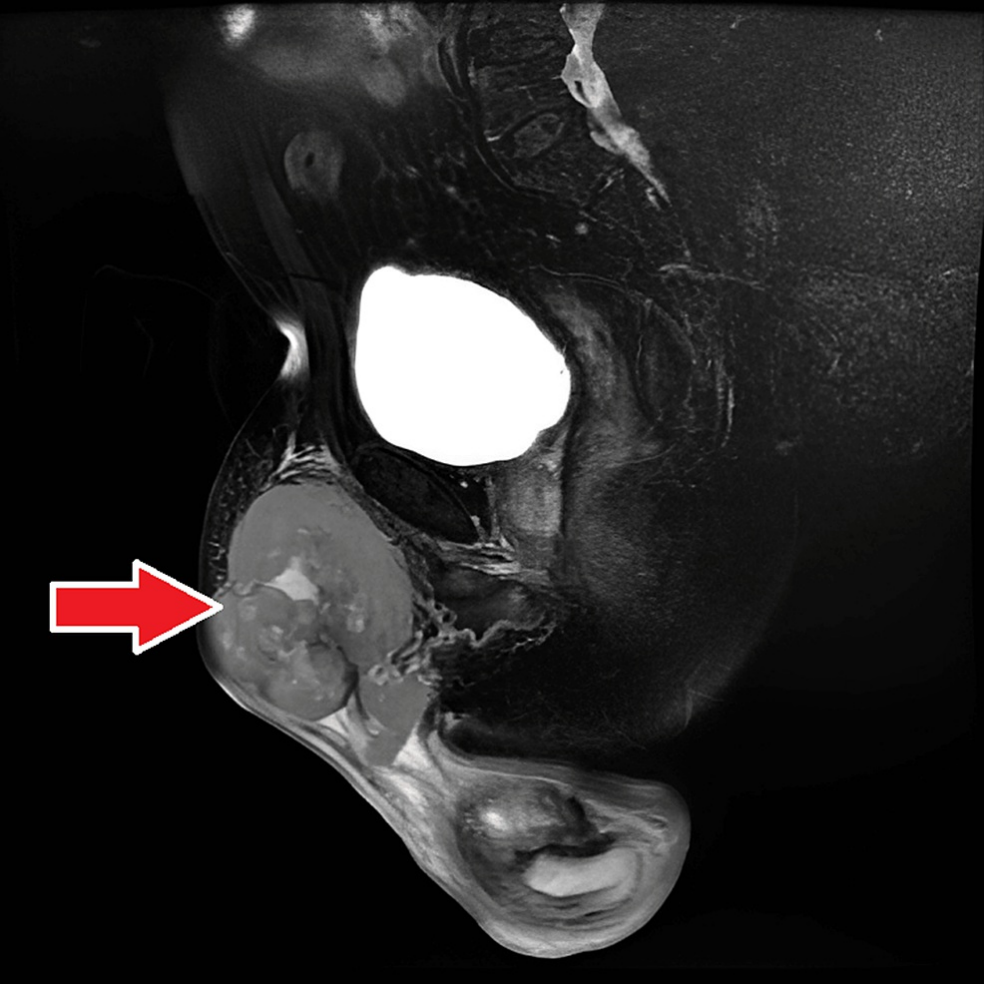
MRI - sagittal T2 FRFSE The image demonstrates a sagittal T2 FRFSE sequence isointense heterogenous mass lesion in the right inguinal region; the mass lesion is interspersed by a few hyperintense areas MRI: magnetic resonance imaging; FRFSE: fast recovery fast spin-echo

**Figure 13:**
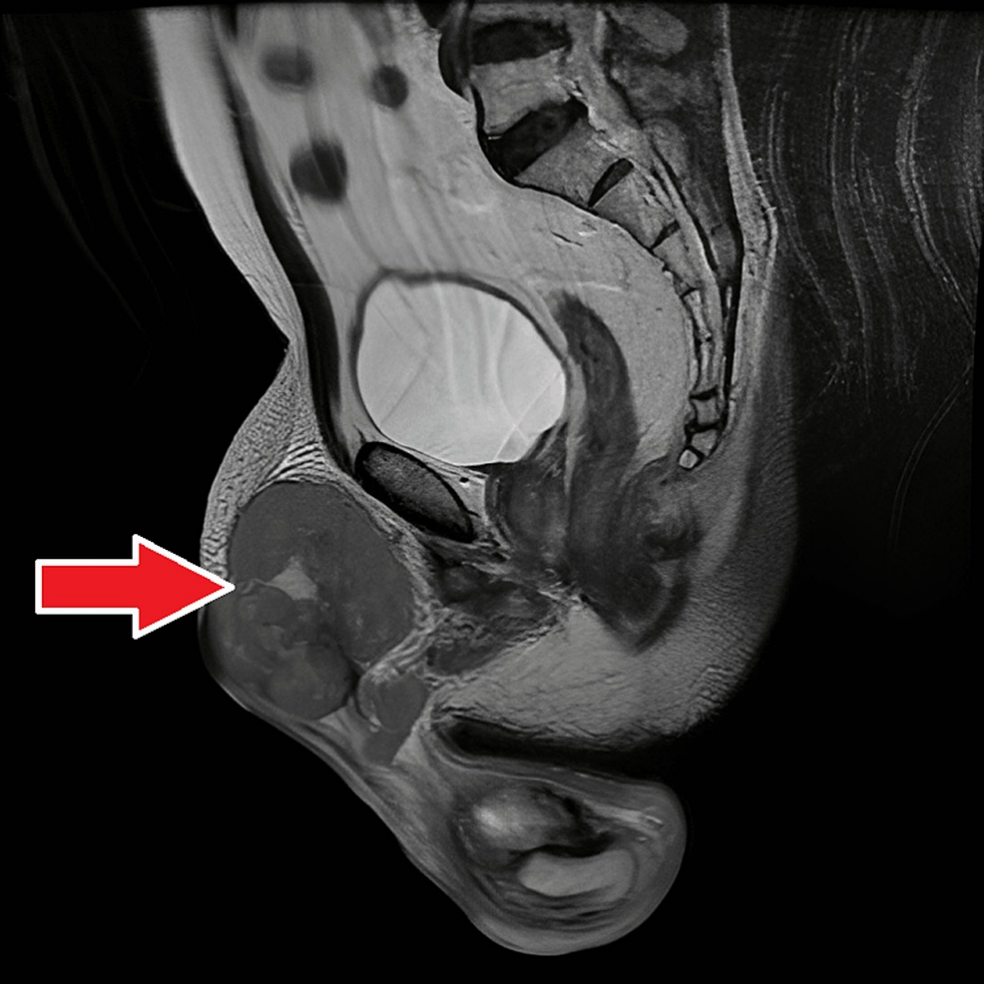
MRI - T2 without fat suppression The image demonstrates sagittal T2 without fat suppression sequence isointense heterogenous mass lesion in the right inguinal region; the mass lesion is interspersed by a few hyperintense areas MRI: magnetic resonance imaging

**Figure 14:**
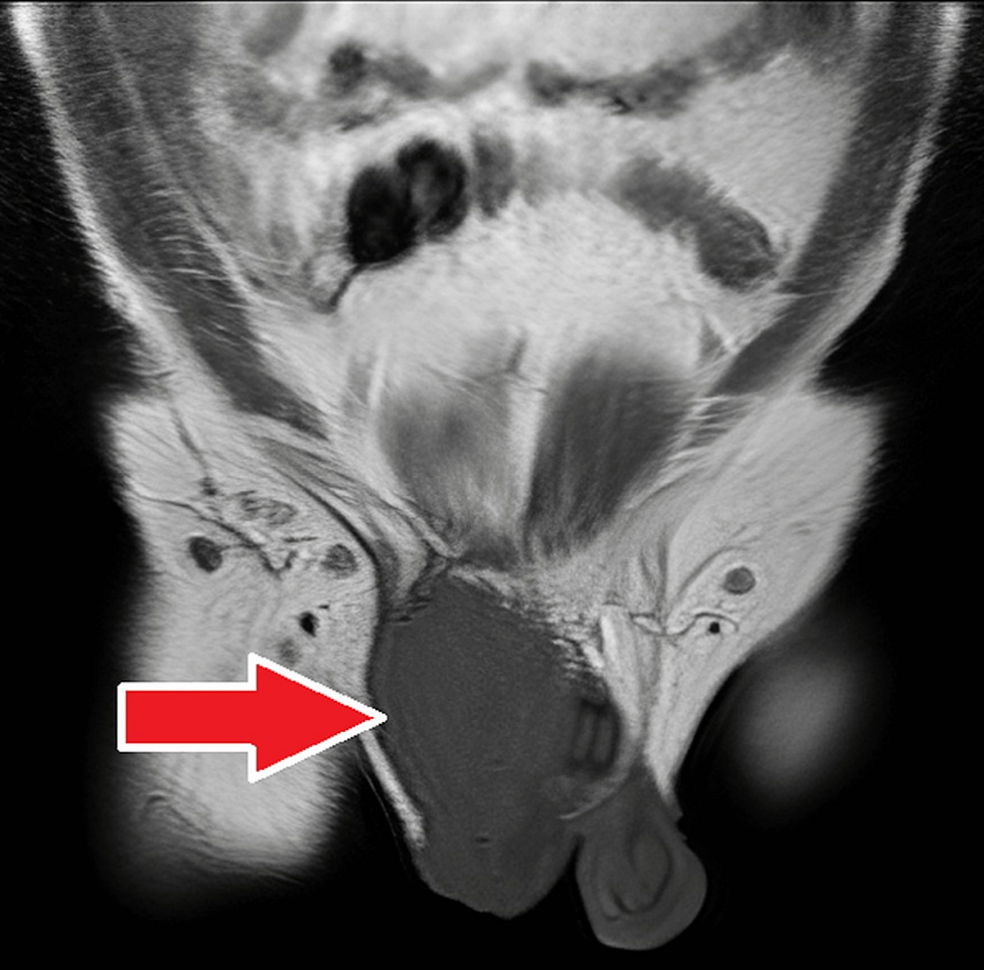
MRI - coronal section of the pelvis The image demonstrates a T1-weighted coronal section of the pelvis that shows diffusely hypointense relatively well-defined mass over the right inguinal region extending into the ipsilateral scrotal sac; contralateral testis and hemiscrotum appear normal MRI: magnetic resonance imaging

**Figure 15:**
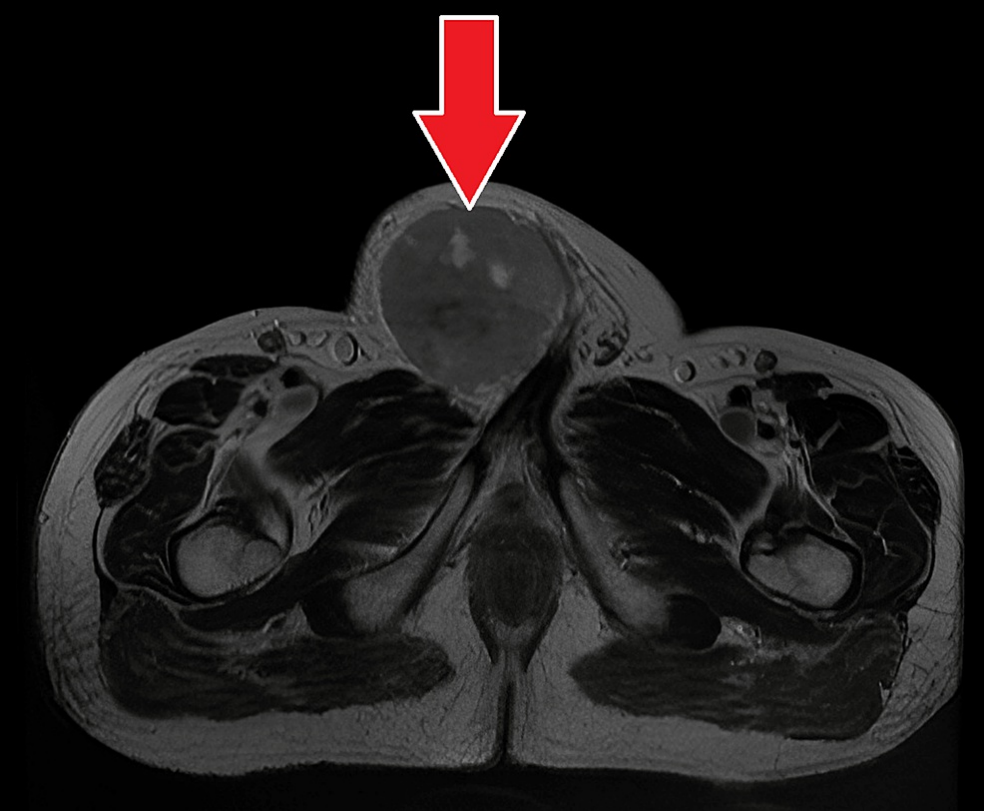
MRI - T2-weighted axial section of the pelvis The image demonstrates a T2-weighted axial section of the pelvis at the level of the inguinal canal; it shows a relatively well defined predominantly hypointense rounded lesion in the right inguinal canal with interspersed areas of hyperintense specks MRI: magnetic resonance imaging

After seven months, the patient returned with swelling over the right inguinal region, which had progressively increased in size. It was examined by the surgeons, which revealed a hard mass of size almost 9 x 6 cm in the right inguinal region; the patient was advised to undergo an MRI of the pelvis along with a CT of the abdomen and chest. The imaging revealed a lobulated, predominantly long TE/TR and short TE/TR sequence intermediate intense lesion measuring 8.5 x 7.0 x 6.0 cm, noted in the right inguinoscrotal region, The lesion was inseparable from the righty cord structures at the root of the scrotum with features of encasement (Figures 12-15).

**Figure 16:**
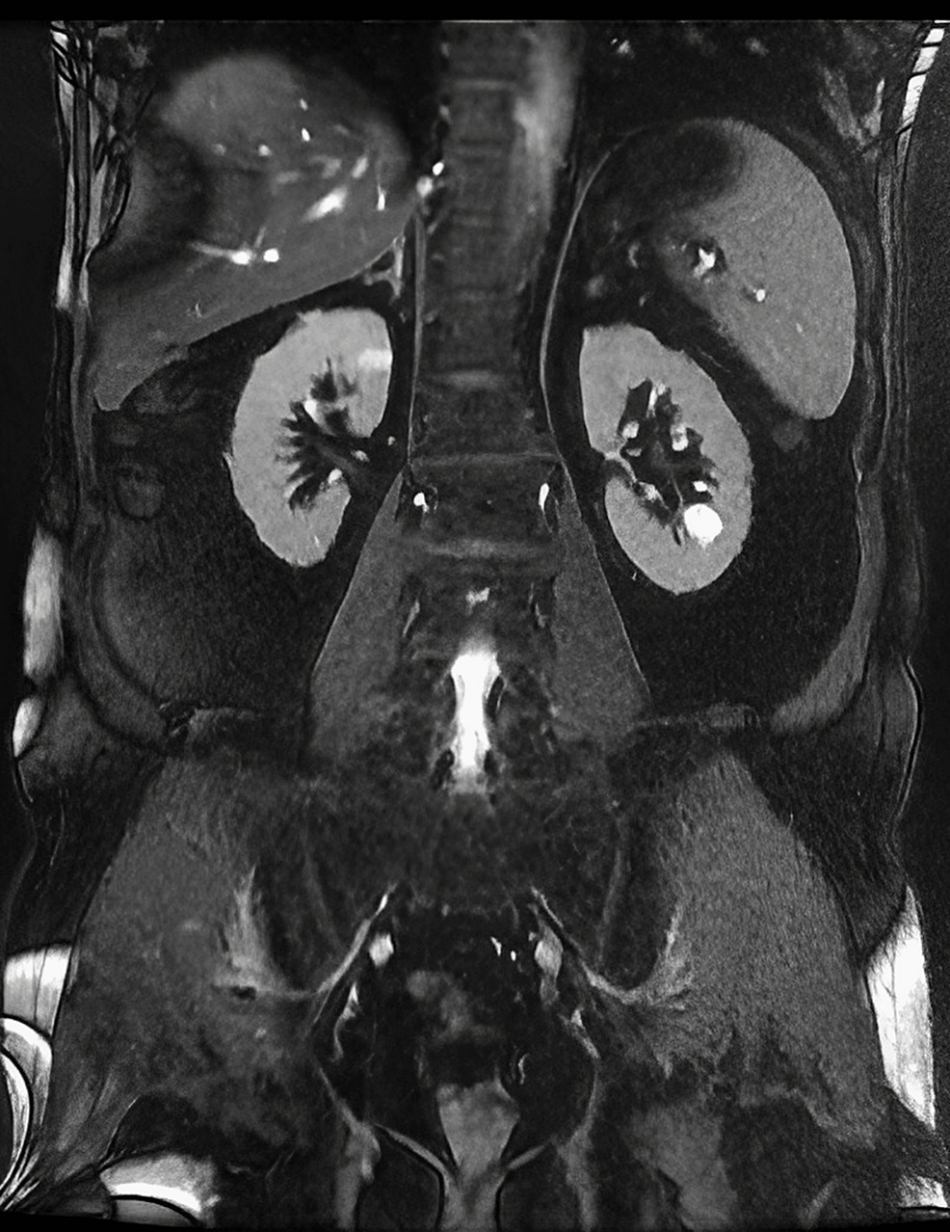
MRI - abdomen Screening of the abdomen using MRI T2-weighted images demonstrates no other discrete lesions noted elsewhere MRI: magnetic resonance imaging

**Figure 17:**
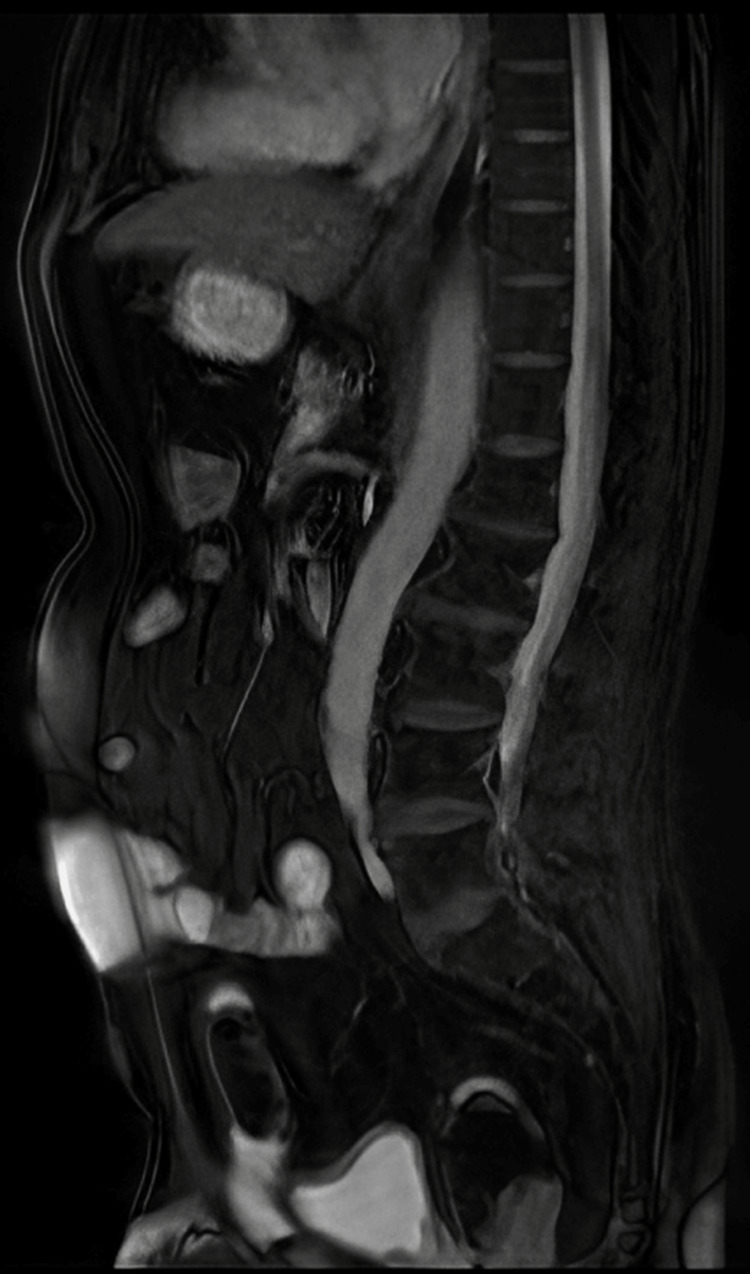
MRI - spine Screening of the spine using MRI T2-weighted images demonstrates no other discrete lesions noted elsewhere MRI: magnetic resonance imaging

**Figure 18:**
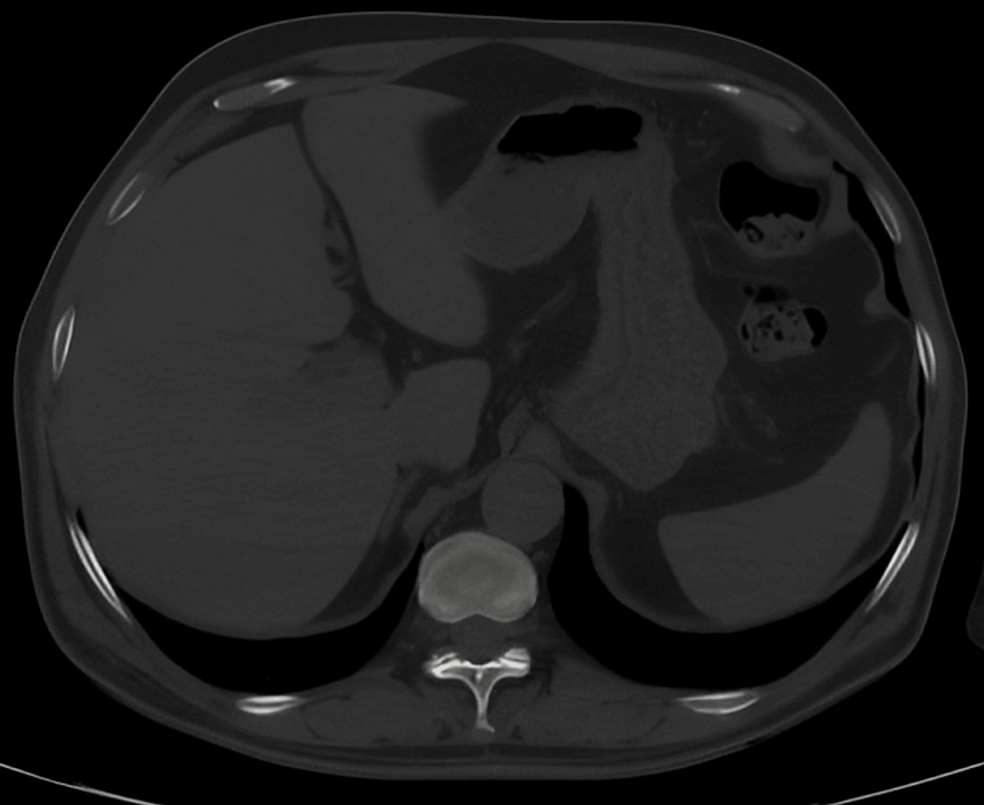
CT - abdomen CT screening of the abdomen axial section showed no discrete lesions in the liver and spleen CT: computed tomography

Small nodules of similar intensity were noted within the scrotal sac. Testis was not recognized separately, consistent with surgical history. The overlying subcutaneous fat plane was inflamed. Screening imaging using both MRI and CT (Figures 16-18) of the abdomen and spine revealed no significant pathology.

The patient then underwent high ligation of the cord structures with mass excision and superficial lymph node resection, and the samples were sent for histopathological correlation, which revealed a monotonous population of cells arranged in sheets and in trabeculae. The cells had eosinophilic to clear cytoplasm with pleomorphic vesicular nuclei with some showing irregular nuclear membranes with one to three prominent nucleoli with high mitotic rate, suggestive of recurrence of diffuse B-cell lymphoma. Furthermore, the patient was advised to undergo high-dose chemotherapy with autologous stem cell transplantation; however, due to its high cost, the patient opted to undergo second-line therapy with chemotherapic agents, which included a combination of ifosfamide + carboplatin + etoposide regimen (ICE regimen) for four cycles with a gap of three weeks between the cycles at the same government-funded higher clinical center.

During the following four months, the patient developed a swelling over the superior aspect of the right shoulder. Fine needle aspiration cytology was undertaken from this site, which revealed atypical pleomorphic cells, consistent with an atypical lymphoproliferative disorder, which suggested dissemination of the disease to the medial end of the right clavicle; following this, the patient was lost to follow-up.

## Discussion

PTNHL is a rare form of primary testicular malignancy that accounts for 1% [3] of all non-Hodgkin's lymphoma cases and 5-10% [3] of all testicular malignancies. PTNHL typically involves men of older age, mostly between the sixth and eighth decades of life [3]. The tumor demonstrates both metachronous and synchronous involvement with the latter type representing the least level of involvement. There is a tendency to disseminate to regions of the body such as the central nervous system and lungs, with the involvement of the pleura and Waldeyer's ring [4].

Spermatic cord lymphoma is an unusual variety of lymphoma and a rare form of primary spermatic cord malignancy. It accounts for 1.6% of spermatic cord tumors [1,2]. Most of these cases come under the umbrella of diffuse large B-cell non-Hodgkin’s lymphoma, which is the most common histological type [2], with other types including Burkitt and follicular variants. Mid to old-aged men are predominantly affected by this variant of malignancy [1,5-6].

Primary spermatic cord lymphoma is a very rare variant of non-Hodgkin’s lymphoma. It is a lethal form of lymphoma that causes detrimental effects even in the early stages of the disease progression. Gonadal lymphoma is rare [3], and the testis is more commonly involved in this variant. Primary involvement of cord structures is by far the rarest form of involvement. Primary testicular lymphoma and primary spermatic cord lymphoma share most of the features, which are similar in nature, and hence most of the imaging findings also overlap with each other.

The patient commonly presents with a painless mass on either side. The mass is seen to be inseparable from the testis and palpated as firm in consistency. Other symptoms include weight loss, loss of appetite, and night sweats, as well as an associated hydrocele in almost half of the cases [7]. Sonography of the lesion shows a mixed echogenic lesion that is predominantly hypogenous in echotexture [8] and appears hyperaemic [8], which is demonstrated by increased vascularity on Doppler. Based on the appearance, a differential diagnosis would be seminoma; however, based on the patient's age, this can be ruled out.

In CT, the lesion generally appears as a heterogenous mass, which is predominantly hypodense and enhances heterogeneously. In post-contrast studies, the lesion mostly replaces the testis and epididymis parenchyma and is usually associated with a hydrocele. CT is further used to visualize para-testicular spaces and staging for which the Arbor classification is used [6]. MRI shows hypointense signals in the lesion on both long and short TE/TR sequences [5,9], with a moderate to strong diffusion restriction on diffusion-weighted sequences and subtle enhancement [9] after contrast administration.

The treatment usually includes orchidectomy, which is both diagnostic and therapeutic, followed by chemotherapy and radiotherapy. A combined approach can also be undertaken [10]. Prophylactic radiation exposure to the ipsilateral testicle and central nervous organs can be considered [10]. However, it has been shown that relapse of the disease can occur following treatment. The contiguous involvement of the spermatic cord demonstrates the unique feature of this case, where an additional involvement of pathology at the medial end of the right clavicle indeed further adds to its unique characteristic.

Further differentials can include secondary involvement of testis, following widespread involvement of non-Hodgkin lymphoma, as well as epididymo-orchitis; however, this can be excluded since the patient had no other typical associated symptoms of epididymo-orchitis like pain and fever. Although most of the cases had pain as an associated symptom, a few cases of chronic epididymo-orchitis can present as an infiltrative mass of the scrotum, which is very difficult to differentiate from PTNHL. In this case, orchidectomy is undertaken for diagnosis confirmation, whereas in other differentials such as germ cell tumors, it often invades the retroperitoneal group of lymph nodes. This presentation was absent in our case; in addition, germ cell tumors are associated with elevated serum human chorionic gonadotrophin and alpha-fetoprotein, which is not seen in the case of PTNHL.

## Conclusions

Testicular cancer is commonly considered to have a good prognosis, with the advent of modern medical applications such as precise surgical excision, in addition to high-quality/detailed imaging modalities and advanced therapies such as chemo/radiotherapy. In our case, there was a significant relapse rate. Thanks to advanced imaging techniques, early detection is possible and the disease can be differentiated from other potential conditions, and the extent of the disease can be assessed so that the outcome can be favorable. Radiologists play an important role in achieving favorable outcomes for the patient. It is thus essential that radiologists are aware of the various differentials for the painless inguinoscrotal mass, and they should consider PTNHL as a differential when assessing an elderly male patient with a unilateral painless swelling.
